# The Effect of Emotional Labor on Psychological Well-Being in the Context of South Korean Firefighters: The Moderating Role of Transformational Leadership

**DOI:** 10.3390/bs14030167

**Published:** 2024-02-23

**Authors:** Jaeyoung Lim, Kuk-Kyoung Moon

**Affiliations:** 1 Department of Public Administration and Social Welfare, Chosun University, Gwangju 61452, Republic of Korea; jaeyounglim@chosun.ac.kr; 2 Department of Public Administration, Inha University, Incheon 22212, Republic of Korea

**Keywords:** surface acting, deep acting, psychological well-being, transformational leadership, firefighters

## Abstract

Drawing on insights from the conservation of resources theory and the job demands–resources theory, our study investigates the association between two types of emotional labor—surface and deep acting—and the psychological well-being of firefighters. In addition, it investigates the moderating effect of transformational leadership within this context. To this end, this study utilizes ordinary least squares models to analyze survey data from 1453 firefighters in Gyeonggi-do, South Korea’s largest province by population. The findings reveal a negative association between both types of emotional labor and the psychological well-being of firefighters. The study further demonstrates that transformational leadership mitigates the adverse effects of surface acting on psychological well-being. Our research indicates that transformational leadership plays a pivotal role in replenishing lost emotional resources, thereby enhancing the mental and emotional health of those engaged in demanding roles such as firefighting and emergency medical services. Accordingly, the study highlights a vital strategy for maintaining the psychological well-being of firefighters.

## 1. Introduction

Scholarly research in public mental health has shown that firefighters frequently face high-stress and dangerous situations in their work [1,2]. In South Korea, firefighters, who are government employees, undertake critical tasks including fire suppression, emergency rescue, and disaster management [2]. These duties expose them to physical dangers, such as exposure to harmful smoke, extreme heat, and loud noises, as well as to the risk of experiencing traumatic events that may lead to psychological disorders [3]. Despite undergoing rigorous and comprehensive training to adeptly manage emergencies and challenging situations, they remain vulnerable to psychological distress, a situation significantly exacerbated by stressful events such as the COVID-19 pandemic [4]. Given the adverse effects of such occupational stress on crucial work-related outcomes, including job satisfaction, organizational commitment, and job performance, firefighting organizations must identify the factors that shape psychological well-being and develop effective strategies for managing their employees’ psychological health [3,5].

This study highlights emotional labor as a key factor associated with psychological well-being [6,7,8,9], given the varied emotional challenges firefighters face in their roles [1,4]. Emotional labor typically involves two aspects: surface acting, wherein effort is made to mask or feign actual emotions, and deep acting, where the aim is to genuinely feel and display emotions that align with organizational expectations [10]. The conservation of resources (COR) theory illustrates that surface acting can lead to resource depletion and stress owing to mental overexertion, whereas deep acting can generate rewards and positive stress through beneficial interactions with the public [11,12]. From this perspective, a negative correlation between surface acting and psychological well-being but a positive correlation between deep acting and psychological well-being may be hypothesized.

This study also examines the role of human resource practices, particularly those informed by the job demands–resources (JD-R) theory [12], in moderating the effect of emotional labor on psychological well-being [13,14]. It highlights the importance of contextual factors beyond direct emotional labor effects, suggesting that job resources can mitigate the negative impacts of emotional demands [13,15]. The research focuses on transformational leadership as a crucial moderating factor, proposing that leaders who support their firefighters’ mental and emotional health enable them to replenish their emotional resources [16]. Transformational leadership is seen as key to helping firefighters manage the emotional challenges of their work and bridge the gap between their genuine and required emotions when interacting with the public. Thus, transformational leadership is expected to not only alleviate the negative effects of surface acting but also amplify the potentially beneficial effects of deep acting on psychological well-being [17].

Our study makes pivotal contributions to organizational psychology and management, enhancing both theoretical understanding and practical applications, in two key areas. First, it sheds light on the complex dynamics of emotional labor among firefighters, specifically focusing on how surface acting and deep acting are associated with their psychological health. This aspect is crucial, as firefighters regularly face emotionally taxing situations requiring regulation; nevertheless, research on how these efforts affect their well-being has been scant. Our findings underscore the importance of addressing emotional labor in firefighting, offering valuable insights for both individual firefighters and their organizations. Second, the study broadens existing knowledge by exploring how transformational leadership plays a moderating role in the interplay between emotional labor and psychological well-being. We propose that transformational leadership serves as an essential resource and is positively associated with the way emotional labor relates to well-being. This aspect of the study not only advances theoretical frameworks but also provides practical guidance. It suggests that with robust support from transformational leaders, firefighters are better equipped to handle the emotional demands of their work, highlighting the critical role of leadership in fostering a supportive work environment.

In the subsequent section, we explore the prevailing literature on emotional labor and transformational leadership, employing the COR and JD-R theories to underpin the hypotheses of our study. Next, we provide a detailed explanation of the data and variables incorporated into our model as well as a comprehensive account of our findings. Finally, we conclude this work by highlighting the implications of our results, addressing any limitations, and outlining potential avenues for future research.

## 2. Emotional Labor and Psychological Well-Being among Firefighters

Recent studies in organizational sustainability have increasingly spotlighted emotional labor and its profound impact on employee well-being [18,19]. Emotional labor involves managing one’s emotions to produce appropriate facial and bodily expressions in public contexts [10,20]. This concept underscores a critical distinction between humans and machines: humans are innately capable of feeling, expressing, and controlling emotions [21,22]. The way employees manage emotional labor significantly affects various work-related outcomes, including burnout, job dissatisfaction, depression, and memory issues [23]. These effects are particularly notable in high-stress professions such as firefighting, where individuals often encounter situations requiring emotional responses that may not align with the professional demeanor expected by their organizations [1,2,24].

Research on emotional labor primarily focuses on two aspects: surface acting and deep acting [25,26]. Surface acting involves the simulation of emotions that are not genuinely felt and the suppression of actual emotions to alter the emotional expression shown to others, such as customers or the public [10]. This usually occurs when employees conceal their true emotions or display emotions they do not actually feel, known as the “faking in bad faith” strategy ([27], p. 32). The strategy involves disguising or modifying internal negative feelings for an outward, superficial expression. Conversely, deep acting represents a different facet of emotional labor, where employees strive to genuinely feel the emotions required by their role, allowing these authentic emotions to be associated with their external behavior [26]. This process, known as the “faking in good faith” strategy ([27], p. 32), involves aligning one’s internal emotional state with the emotions an organization expects, leading to a more genuine and consistent emotional expression.

Drawing from the COR theory, this study proposes a positive correlation between surface acting and psychological well-being in firefighters. The COR theory suggests that individuals are engaged in the active process of acquiring, maintaining, and safeguarding the resources that are crucial to their well-being [11,28]. It highlights the dynamic nature of resource management, where individuals invest in resources to prevent potential losses, recover from past losses, and acquire new resources [29]. The COR theory recognizes that the psychological impact of resource loss is typically more profound and enduring than the satisfaction of gaining resources [30,31]. Thus, the distress associated with resource loss often surpasses the pleasure of resource gain. Negative emotional outcomes including burnout and job stress primarily arise in situations involving the threat of resource loss, actual resource loss, or inadequate resource gain following investment [32]. For instance, when firefighters encounter emotionally demanding job tasks, they utilize valuable emotional resources such as affection and optimism, anticipating organizational rewards in return. If they feel that the anticipated rewards are of lesser value than the emotional resources they have expended, they view it as a resource drain, which often results in negative outcomes in their work performance [32,33]. On the other hand, if the rewards are perceived to be more significant than, or to surpass, the emotional resources used during their work, they see this as a net increase in regained resources, which then fosters positive attitudes or behaviors at work [11].

Applying the COR theory to the dynamic between emotional labor and job stress shows that surface acting, which involves feigning and suppressing genuine emotions, requires substantial psychological energy and attention, leading to the depletion of valuable emotional resources. As identified by Gross [34], this manipulation of emotional expression demands deliberate and considerable self-control, consuming cognitive resources during emotion regulation and resulting in a net resource loss [35]. Surface acting may thus be associated with a diminished sense of authenticity in firefighters, impede the development of rewarding social relationships, be correlated with higher job dissatisfaction, and be linked to lower emotional commitment to work. Moreover, firefighters engaging in surface acting frequently experience emotional dissonance—a discrepancy between internal feelings and external expressions—which progressively drains their cognitive and motivational resources [36]. This depletion amplifies psychosocial risks such as burnout, exhaustion, and cynicism, escalating job stress [37]. In contrast, deep acting, characterized by the alignment of one’s emotions with those favored by the organization, is hypothesized to reduce psychological well-being. Firefighters practicing deep acting might receive public support and appreciation for their authenticity, enhancing their sense of achievement and performance [38]. Although deep acting is effort-intensive, it replenishes resources by reducing the discrepancy between internal emotions and external expressions, thus mitigating the negative emotional and psychological effects of demanding work experiences [39,40].

Indeed, in their study of Canadian employees, Brotheridge and Grandey [29] found that surface acting is positively associated with emotional exhaustion and depersonalization but negatively associated with personal accomplishment. The results also showed that employee deep acting has a positive impact on personal accomplishment. Similarly, in the context of the hospitality industry in Taiwan, Wang et al. [41] provided empirical evidence that surface acting is associated with decreased job satisfaction and mental health owing to its effort-intensive nature and the creation of emotional dissonance. Conversely, deep acting is associated with higher job satisfaction and improved mental health, as it fosters an emotional state where felt and displayed emotions are congruent. These findings indicate that surface acting, a method of emotion regulation focusing on the outward expression of emotions, demands additional mental effort from employees, potentially depleting their emotional energy and resilience. This process can negatively affect the psychological well-being of employees by elevating stress levels and diminishing their job satisfaction. Conversely, deep acting, which is a proactive approach to emotion regulation that targets the actual experience of emotions, encourages employees to actively engage in feeling more positive during customer interactions. This approach is beneficial for enhancing mental and emotional well-being. Based on these arguments and previous empirical evidence, the following hypotheses are formulated:

**Hypothesis 1.**  *Surface acting is negatively related to psychological well-being*.

**Hypothesis 2.**  *Deep acting is positively related to psychological well-being*.

## 3. The Moderating Effect of Transformational Leadership

Bass [42] identified four distinct behaviors exhibited by transformational leaders: (a) idealized influence, which involves providing followers with a vision and emotional connection along with a high level of trust in their leaders; (b) inspirational motivation, where leaders communicate an enticing future vision and demonstrate the path to achieve organizational objectives; (c) intellectual stimulation, which encourages team members to think creatively and imaginatively; and (d) individualized consideration, focusing on acknowledging and catering to the unique needs of individuals through mentoring and feedback. These behavioral aspects enable transformational leaders to mold employee behaviors and attitudes for effective organizational operation [43,44,45]. Transformational leadership can motivate employees to exceed expectations and undertake voluntary actions by aligning their interests with broader organizational goals, superseding personal interests.

More importantly, transformational leadership, as a psychological resource of an organization, may moderate the relationships between the two forms of emotional labor and psychological well-being [16,46]. The JD-R theory, central to the study of occupational psychology, categorizes the work characteristics present in various professions into two essential types: job demands (JD) and job resources (JR) [47]. Job demands encompass the aspects of a job, whether physical, social, psychological, or organizational, that require continuous physical or mental effort [48]. This effort often leads to outcomes such as increased work pressure and the risk of burnout [49]. Job resources are defined as the elements of a job, in any of these domains, that aid in achieving work objectives, reduce the pressure of job demands, and contribute to individual and professional development [48]. Examples of job resources include performance feedback, involvement in decision-making, satisfaction of employees’ personal needs, and supervisory dedication to employee welfare [16]. The JD-R model proposes that high job demands can lead to stress, resulting in employee fatigue and decreased job performance [50]. However, the availability and effective use of various job resources can mitigate the adverse effects of excessive job demands, thus alleviating the stress caused by emotional demands or work-related pressure [48].

The JD-R theory posits that transformational leaders play a significant role in modulating the intensity of job demands and the availability of job resources. This strategic adjustment is key to maintaining low levels of employee burnout while fostering high levels of engagement at work, which is considered critical in bolstering mental and emotional health [16]. Echoing this sentiment, Schaufeli [51] found that the responsibility of leaders extends to ensuring a harmonious balance of job demands and resources for their team members with the aim of preserving their health, motivation, and productivity. These perspectives collectively underline the essential role of transformational leadership within the JD-R framework in ensuring a balance between job demands and resources with the ultimate goal of promoting employee engagement and increasing their overall psychological well-being [52]. Given that surface acting leads to resource depletion among employees, who then seek alternative job resources to offset this deficit, transformational leadership emerges as an essential external energy source that aids in emotional recuperation amidst emotional labor tasks [16].

Transformational leaders effectively realize this by articulating a vision that is not only attainable but also replete with new opportunities; this resonates with the JD-R theory, which posits that such a vision acts as a powerful motivational agent, invigorating employees [53]. Transformational leaders personalize their approach, offering feedback, coaching, and advice that are congruent with each employee’s unique skills as well as acknowledging their achievements. This strategy not only fosters a positive work environment but also amplifies the employees’ emotional energy, thus mirroring the JD-R model’s emphasis on balancing job demands with adequate resources [16]. Furthermore, the transformational leadership approach of empowering subordinates, clarifying goals, and striving for the highest outcomes instigates intellectual development [16,46]. It motivates employees to view challenges through a lens of innovation and to devise creative solutions. This aspect of transformational leadership is particularly effective in enhancing employees’ eagerness to learn and adapt to new skills and knowledge [16,46]. For instance, firefighters who feel that their leaders exhibit genuine empathy and supportive guidance can supplement potential resource depletion caused by surface acting and deal with highly intensive job demands, leading to improved psychological well-being [54]. This may imply that transformational leadership helps mold firefighters performing surface acting into resilient and emotionally well-adjusted individuals. In the same vein, firefighters engaging in deep acting under the guidance of transformational leaders might perceive an enhancement in their willpower and psychological vitality, which facilitates their investment in deep acting amidst emotionally charged service interactions, thereby fostering excellence in their professional capacities [52]. Accordingly, transformational leadership significantly contributes to motivating firefighters who utilize deep acting techniques, instilling in them a zeal for sustaining and elevating their psychological and emotional well-being [55].

Despite the theoretical discussions of the moderating effects of transformational leadership, empirical research exploring how this leadership style is associated with the relationship between emotional labor and employees’ psychological well-being is lacking. Although many studies have focused on the moderating roles of perceived organizational support, psychological capital, gender, and personality in the context of emotional labor and its work-related outcomes [13,56,57,58], the specific impact of transformational leadership in creating an environment that alleviates the negative effects of emotional labor, especially in terms of surface and deep acting, is not well understood. This research gap underscores the necessity for empirical studies focusing on how transformational leaders can counteract the detrimental effects of emotional labor to improve employee psychological well-being. Therefore, we propose the following hypotheses:

**Hypothesis 3.**  *Transformational leadership moderates the negative relationship between surface acting and psychological well-being such that the relationship is weaker when transformational leadership is higher rather than lower*.

**Hypothesis 4.**  *Transformational leadership moderates the positive relationship between deep acting and psychological well-being such that the relationship is stronger when transformational leadership is higher rather than lower*.

## 4. Methodology

### 4.1. Data Sources and Sample

In this research, survey responses were collected from firefighters in Gyeonggi-do, South Korea’s largest province by population. These firefighters, who are public servants, have met the requirements of civil service examinations and work under the auspices of the central government. Gyeonggi-do has 9686 street-level firefighters who are spread across 35 fire stations and engage in extinguishing dangerous fires and conducting rescue operations. The job roles and authorities of these firefighters are structured within a seven-tier job-grade hierarchy ranging from firefighter (Grade 1) to fire chief (Grade 7). The responsibilities vary across these grades; for instance, entry-level firefighters primarily engage in frontline fire suppression and rescue, whereas those at the topmost level oversee and manage the fire station, with responsibilities including training, performance evaluation, and duty assignment.

Given the distinct and well-defined job-grade hierarchy in fire stations, a quota sampling method based on job grades was employed to enhance the external validity of the study results. This approach ensured representation from each job grade, although it did not fully capture the diverse demographic profiles of all firefighters in Gyeonggi-do, such as gender and educational background. The survey was conducted via email from April 6 to April 17, 2020. After discarding invalid responses, such as those with uniform answers or excessive missing data, a total of 1578 valid responses were obtained, representing a 16.27% response rate. All survey items, excluding demographic questions, were rated on a Likert-type scale ranging from 1 (strongly disagree or very unlikely) to 5 (strongly agree or very likely).

### 4.2. Dependent Variable

The General Health Questionnaire (GHQ-12) is a widely utilized tool designed to assess psychological well-being [59,60]. This concise questionnaire comprises 12 questions that effectively gauge an individual’s mental health status, focusing on areas such as general happiness, anxiety, social dysfunction, and self-esteem. The GHQ is characterized by its approach of asking respondents how they have psychologically felt over the past month, thereby identifying problems in their current state. The questionnaire is structured to discern changes in normal psychological functioning, thus making it particularly adept at identifying signs of distress or potential mental health issues. To measure psychological well-being, we utilized the following survey items developed by Goldberg and Williams [61]: (1) “Have you recently been able to concentrate on what you’re doing?” (2) “Lost much sleep over worry?^®^” (3) “Felt you were playing a useful part in things?” (4) “Felt capable of making decisions about things?” (5) “Felt constantly under strain?^®^” (6) “Felt you couldn’t overcome your difficulties?^®^” (7) “Been able to enjoy your normal day-to-day activities?” (8) “Been able to face your problems?^®^” (9) “Been feeling unhappy and depressed?^®^” (10) “Been losing confidence in yourself?^®^” (11) “Been thinking of yourself as a worthless person?^®^” (12) “Been feeling reasonably happy, all things considered?” Here, “^®^” indicates reverse coding. Respondents answered all questions on a four-point Likert scale—1 (not at all), 2 (same as usual), 3 (rather more than usual), and 4 (much more than usual)—reflecting varying degrees of psychological well-being. With reverse coding, lower scores generally indicate poorer psychological well-being, whereas higher scores indicate better psychological well-being.

### 4.3. Independent Variables

In the current study, measurement scales for surface and deep acting were developed using the same six items employed by Brotheridge and Lee [38]. A sample item for measuring surface acting is “I resist expressing my true feelings”. A sample item for measuring deep acting is “I make an effort to actually feel the emotion that I need to display to others”. The items were assessed on a Likert-type scale, which varied from 1 (strongly disagree) to 5 (strongly agree).

### 4.4. Moderating Variable

We used the same five items developed by House [62] to measure transformational leadership. Sample items for measuring transformational leadership include “My supervisor communicates his or her vision of the future” and “My supervisor challenges me to think about old problems in new ways”. The evaluation was based on a five-point Likert scale ranging from 1 (strongly disagree) to 5 (strongly agree).

### 4.5. Controls

Although studies on the association between various demographic characteristics and psychological well-being have yielded inconsistent results, certain systematic reviews have identified organizational fairness, organizational support, and demographic characteristics as significant correlates of psychological well-being [60,61]. Organizational fairness is defined as an employee’s perception of the overall fairness in the organization’s reward system [63]. Four items developed by Leventhal [64] were used to measure organizational fairness. Sample items include “Does your outcome reflect the effort you have put into your work?” and “Is your outcome appropriate for the work you have completed?” Organizational support is defined as employees’ perception of the extent to which their organization values their contributions and cares about their well-being [65]. We measured organizational support using four items adapted from Eisenberger et al.’s [65] survey of perceived organizational support. Sample items include “My organization genuinely cares about my well-being” and “My organization is willing to help me fully utilize my abilities to perform my job”. All items for organizational fairness and organizational support were measured on a Likert-type scale ranging from 1 (strongly disagree) to 5 (strongly agree). Our research considered various personal demographic attributes of the participants that might affect their psychological well-being. These included gender (1 for female, 0 for male), age categories (1 for 20–29 years, 2 for 30–39 years, 3 for 40–49 years, 4 for 50 years or older), educational level (1 for high school, 2 for college, 3 for a bachelor’s degree, 4 for graduate school), job grade (ranging from 1 for Grade 1 to 7 for Grade 7), length of tenure (1 for less than nine years, 2 for 10–19 years, 3 for 20–29 years, 4 for more than 30 years), and marital status (1 for married, 0 for unmarried). Table 1 presents the descriptive statistics of the variables used in this study.

### 4.6. Measurement Reliability and Validity

We evaluated the reliability of our measures through confirmatory factor analyses using structural equation modeling (SEM), as illustrated in Table 2. Our hypothesized six-factor model, encompassing psychological well-being, surface acting, deep acting, transformational leadership, organizational fairness, and organizational support, demonstrated an optimal fit with the data. The model’s fit was assessed with several indicators: the root mean square error of approximation (RMSEA) and the standardized root mean square residual (SRMR) values were 0.06 and 0.07, respectively, both below the preferred threshold of 0.08. In addition, the comparative fit index (CFI) and the Tucker–Lewis Index (TLI) stood at 0.92 and 0.91, respectively, surpassing the 0.9 benchmark and suggesting a good model fit.

Factor loadings for all items significantly exceeded 0.50, ranging from 0.50 to 0.94, supporting the distinctiveness of the measures for the four latent variables. Composite reliabilities for these variables were confirmed with Cronbach’s alpha, yielding coefficients ranging from 0.82 to 0.96—well above the recommended 0.70 threshold. To address potential common method variance (CMV) that could artificially inflate the relationships between variables, we conducted Harman’s single-factor test. The primary factor explained only 31% of the variance among the measures, below the 50% threshold commonly associated with significant CMV concerns. Therefore, CMV does not appear to significantly distort the relationships observed in our study.

## 5. Results

Table 3 provides the correlation coefficients, Cronbach’s alpha (α), and average variance extracted (AVE) for all the latent variables. The correlation analysis reveals that the two aspects of emotional labor are negatively correlated with psychological well-being, whereas transformational leadership, organizational fairness, and organizational support are positively correlated with psychological well-being. The Cronbach’s alpha and AVE of the six latent variables are greater than 0.7 and 0.5, respectively, indicating good internal reliability and convergent validity in this study’s sample.

This research investigates the impacts of surface and deep acting on psychological well-being among South Korean firefighters and assesses whether transformational leadership is related to these relationships. For data analysis, we used ordinary least squares (OLS) regression, as our dependent variable was based on summed averages. The hierarchical regression analysis detailed in Table 4 outlines our results. Models 1 and 2 focus on the linear representations of all variables, illustrating how surface and deep acting are directly associated with psychological well-being. In Model 3, we introduce two interaction variables—surface acting combined with transformational leadership and deep acting combined with transformational leadership—to examine how transformational leadership potentially moderates the relationships of both surface acting and deep acting with psychological well-being. In addition, our models utilize the Huber–White sandwich estimator for robust standard errors to address any heteroskedasticity issues.

In terms of control variables, the results of Model 1 indicate that gender is significantly associated with psychological well-being (*β* = −0.060, *p* < 0.05), suggesting that the psychological well-being of female firefighters is lower than that of male firefighters. In the context of South Korean society, where traditional gender roles often allocate a greater share of domestic and childcare duties to women than to men, female firefighters might face the dual challenge of managing these domestic responsibilities while meeting their professional obligations. This juxtaposition of societal expectations and individual roles may exert additional stress on the psychological well-being of female firefighters, resulting in increased challenges for them in achieving a harmonious balance between their work and personal life. Our findings also reveal that age is significantly and negatively associated with psychological well-being (*β* = −0.037, *p* < 0.10), which may be attributable to older firefighters being exposed to more trauma over the course of their work. Marital status is positively associated with psychological well-being (*β* = 0.100, *p* < 0.001) possibly because the presence of a spouse or family member can provide emotional stability when facing daily stress and professional risks. This emotional support can promote psychological well-being. The findings further indicate that organizational fairness (*β* = 0.064, *p* < 0.001) and organizational support (*β* = 0.139, *p* < 0.001) are positively associated with psychological well-being. Fair and supportive environments can lead to higher levels of employee engagement and job satisfaction. When firefighters feel valued and perceive equitable treatment from their organizations, they are more likely to be content with their job, which is positively associated with their mental health. Finally, transformational leadership is positively associated with the psychological well-being of firefighters (*β* = 0.125, *p* < 0.001). Transformational leaders help employees achieve their work and personal goals by inspiring and motivating them—thus improving their psychological well-being and increasing job satisfaction—and by taking an interest in and supporting their personal development and growth. Moreover, transformational leadership is associated with higher levels of employees’ enthusiasm toward and engagement in their work and correlates with building consensus around the organization’s vision and goals, thereby strengthening their sense of purpose and emotional security.

In support of Hypothesis 1, Model 2 reveals that surface acting is negatively associated with firefighters’ psychological well-being (*β* = −0.193, *p* < 0.001). Suppressing emotions and expressing false emotions may require increased emotive effort that leads to negative effects, including increased stress and emotional exhaustion, and thus decreases psychological well-being. However, contrary to our expectations, the results demonstrate a negative association between deep acting and psychological well-being (*β* = −0.024, *p* < 0.05), contradicting Hypothesis 2. This finding suggests that although deep acting may be less demanding than surface acting, it still requires significant effort, leading employees to expend emotional resources. Both surface and deep acting, as emotion regulation strategies, potentially deplete emotional resources. Importantly, our findings show that both have a detrimental effect on psychological well-being, with the association of deep acting being notably less severe than that of surface acting.

The initial models act as essential reference points for our primary models of interest. In Model 3, we incorporated interactions between surface acting, deep acting, and transformational leadership to more thoroughly examine the JD-R theory. Our results indicate that the two interaction terms—surface acting × transformational leadership (*β* = 0.033, *p* < 0.05) and deep acting × transformational leadership (*β* = 0.023, *p* < 0.10)—demonstrate positive coefficients for psychological well-being. This suggests that transformational leadership mitigates the negative impacts of both surface acting and deep acting on psychological well-being. These findings are in alignment with Hypotheses 3 and 4, respectively.

**Figure 1:**
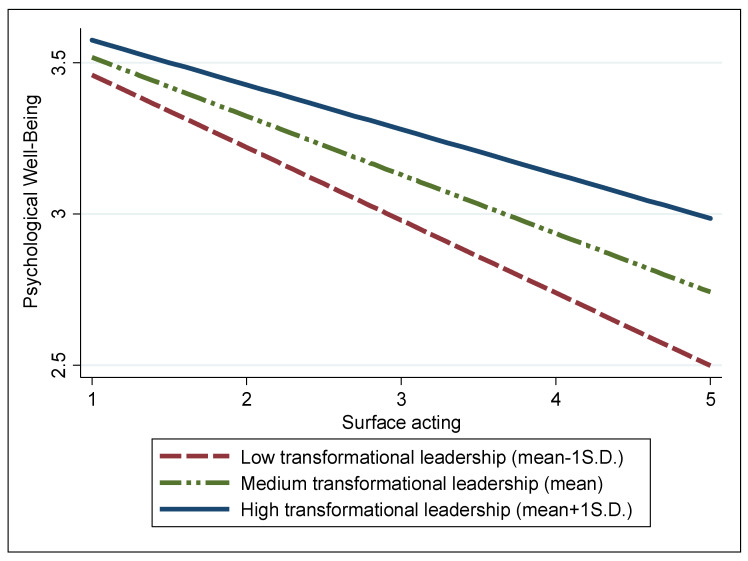
Moderating effect of transformational leadership on the relationship between surface acting and psychological well-being.

**Figure 2:**
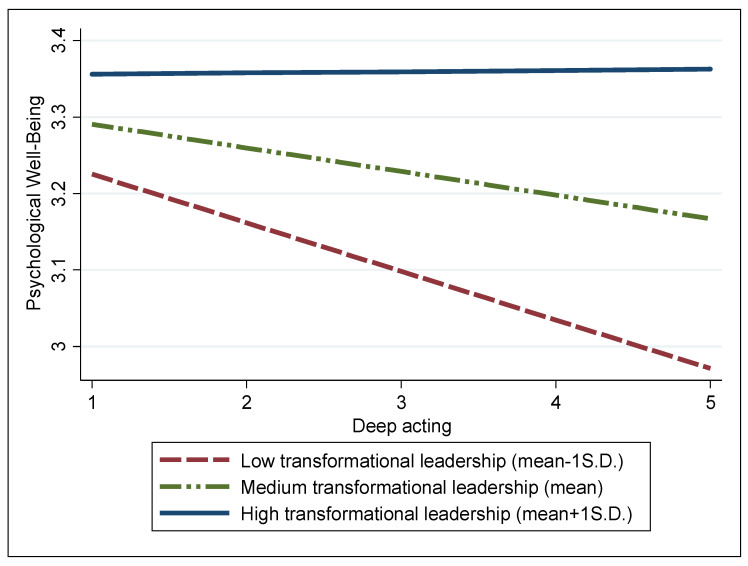
Moderating effect of transformational leadership on the relationship between deep acting and psychological well-being.

To elucidate the interactions between the two types of emotional labor—surface and deep acting—and transformational leadership, we visualized the moderating effects of transformational leadership on the relationships between these emotional dimensions and psychological well-being in Figure 1 and Figure 2, respectively. As anticipated, Figure 1 demonstrates that the detrimental link between surface acting and the psychological well-being of firefighters is notably diminished under high transformational leadership, marked as one standard deviation (S.D.) above the mean (blue solid line). In contrast, this negative relationship intensifies under low transformational leadership, which is one S.D. below the mean (red dotted line). Figure 2 also illustrates that compared with low transformational leadership, high transformational leadership more significantly alleviates the negative association of deep acting with psychological well-being.

## 6. Discussion and Implications

Based on the COR theory, our research investigates how two types of emotional labor are associated with the psychological well-being of firefighters in South Korea. In addition, we explore the moderating role of transformational leadership in these interactions based on the JD-R theory. The results, primarily aligning with the theoretical frameworks illustrated in Figure 1 and Figure 2, suggest that transformational leadership plays a key role in alleviating the negative impacts of both surface and deep acting on the psychological well-being of firefighters. Consequently, this study offers important insights and applications for managing mental health in occupations that involve high levels of stress.

### 6.1. Theoretical Implications

In alignment with the COR theory, our findings indicate that firefighters engaging in surface acting are likely to experience a decline in psychological well-being. This form of emotional labor necessitates significant self-control and deliberate effort, leading to the consumption of cognitive resources during emotion regulation [10] and ultimately resulting in a net loss of resources. Surface acting, characterized by a discrepancy between felt and displayed emotions, may erode a firefighter’s sense of authenticity and hinder the formation of fulfilling social connections. This, in turn, can amplify job dissatisfaction and reduce emotional engagement with their work. The frequent experiences of emotional dissonance associated with surface acting further drain cognitive and motivational resources, contributing to lower levels of psychological well-being. Therefore, our study reinforces the validity of the COR theory as an effective framework for examining the associations between surface acting and psychological well-being.

Contrary to our initial assumptions, our study uncovers a negative association between deep acting and the psychological well-being of firefighters. Deep acting, which involves aligning external expressions with modified internal feelings [27], was anticipated to be less psychologically demanding than surface acting. However, this process may demand considerable cognitive and emotional labor, as it requires the continuous modification of genuine emotions to meet job expectations, leading to cognitive overload and emotional strain that adversely affect well-being [29]. Further, although deep acting involves the alteration of real emotions, it can inadvertently result in a sense of inauthenticity or emotional dissonance [26]. Firefighters who regularly engage in deep acting may face an internal conflict between their true emotions and the emotions they need to display professionally, thus eroding their sense of self-authenticity and further diminishing their psychological well-being. This complexity is further illuminated through the lens of the COR theory, which views deep acting as a resource-depleting activity. That is, the persistent adjustment of emotions to conform to organizational norms not only is demanding, but also exhausts the emotional resources vital for managing other job-related stress and personal challenges. Consequently, this depletion of emotional resources is key to understanding the unexpected detrimental association between deep acting and the psychological well-being of firefighters.

Our research aligns with the principles of the JD-R theory, revealing that transformational leadership is significantly associated with reduced negative impacts of both surface and deep acting on psychological well-being. This finding underscores the vital role of transformational leadership as a dynamic job resource in moderating the complex interplay between the two dimensions of emotional labor and employees’ mental health [53]. Employees in work environments with transformational leaders frequently feel that their leaders are deeply committed to their comprehensive personal and professional development and receive higher levels of supervisory support [16,46]. Moreover, transformational leaders stand out, not only in their ability to inspire and motivate their teams, but also in their skills at challenging employees to push their limits and innovate [16,46]. This creates a culture that values and embraces genuine emotional expression, which is instrumental in fostering employees’ emotional intelligence and resilience. Such leadership enables employees to more adeptly handle workplace stress, thereby improving their overall job satisfaction and commitment. Furthermore, transformational leaders personalize their leadership approach, offering tailored feedback, coaching, and advice that resonate with each employee’s unique skills and strengths while also acknowledging their individual achievements [54]. This personalized leadership approach builds a positive and supportive work environment and significantly enhances the emotional energy of the workforce, perfectly aligning with the JD-R model’s emphasis on balancing job demands with sufficient resources [16,46]. Considering these insights, we anticipate that transformational leadership plays a pivotal role in diminishing the negative psychological outcomes among employees, such as emotional exhaustion and depersonalization, by supplying a range of supportive external resources. This leadership approach emerges as a vital component in promoting a healthier, more resilient workforce.

### 6.2. Practical Implications

Our study findings present pivotal implications for the practical management of emotional labor in high-stress professions such as firefighting. We offer new managerial insights suggesting that increased focus on emotional labor management strategies can significantly improve firefighters’ work outcomes. Understanding the association between emotional labor and firefighters’ emotional health is crucial in today’s demanding work environments, as the effective management of their psychological well-being is integral to organizational performance and sustainability. Our research underlines that an organization’s success and longevity are closely linked to how it manages its employees’ emotional labor. Rather than discarding emotional display rules to prevent surface acting and encourage deep acting, firefighting and emergency medical service organizations should implement comprehensive emotional training programs. These programs should aim to equip employees with a thorough understanding of and skills in emotion regulation strategies and personal resource management, thereby enabling them to skillfully navigate emotionally charged interactions with the public. Such training could include strategies such as situation selection for emotion regulation, situation modification to alter emotional impacts, attentional deployment to manage emotions, and cognitive change to reframe situational appraisals. By incorporating these strategies, firefighters can more effectively regulate negative emotions and adapt their expressions, enhancing their capacity to handle work-related emotional challenges. This approach not only ensures the mental health of individual firefighters but also bolsters the overall effectiveness and resilience of the organizations they serve.

Another recommendation for practitioners, based on our findings, emphasizes the benefits of transformational leadership in mitigating the negative associations of both surface and deep acting with psychological well-being. Transformational leadership, characterized by its inspirational, motivational, and individualized consideration aspects, is notably effective in reducing the drain of emotional resources caused by both forms of emotional labor. Therefore, firefighting and emergency medical service organizations must implement a human resource management strategy focused on nurturing transformational leadership qualities in their managers. First, organizations must highly prioritize fostering transformational leadership skills by providing well-rounded professional development programs specifically designed for leaders. These programs should comprehensively address key leadership competencies. The programs could include effective communication training focused on techniques for delivering clear and motivational messages. Emotional intelligence training is also essential for teaching leaders how to understand and manage their emotions while empathizing with others. Moreover, conflict resolution sessions are crucial for guiding leaders on constructively navigating and resolving workplace disputes. Equally important is team building, which should aim to create cohesive and collaborative teams. Finally, vision setting is vital and involves the development and articulation of a compelling and strategic organizational vision. Altogether, these elements form a robust framework for cultivating effective transformational leadership within an organization. Second, integrating transformational leadership criteria into the performance appraisal of managers is vital. This can be achieved by establishing specific metrics and benchmarks based on transformational leadership behaviors, such as the degree of inspiration provided to teams, the effectiveness of communication, and the ability to foster an innovative and open work environment. Regular feedback sessions should be held to discuss these appraisals, offering managers constructive insights into their leadership style and areas for improvement. Third, establishing mentorship and coaching programs is essential. These programs would allow novice or less experienced leaders to benefit from the wisdom and experience of seasoned leaders. The mentors can share their leadership journeys, challenges, successes, and strategies, providing real-world insights and guidance. Finally, creating an organizational culture that actively supports and values transformational leadership is crucial. This can be achieved by publicly recognizing and rewarding managers who demonstrate these leadership behaviors. For example, a “Leader of the Month” program can highlight individuals who exemplify transformational leadership qualities. In addition, establishing platforms where leaders can showcase their innovative ideas and initiatives encourages a culture of transformational leadership.

### 6.3. Limitations and Future Research

This study’s limitations provide valuable directions for future research. Here, we utilized a cross-sectional self-report survey and subjective measures, potentially leading to CMV. Although Harman’s single-factor test indicated minimal CMV concerns, future studies should employ more objective measures and longitudinal data to enhance causal inference accuracy. In addition, applying latent variable modeling techniques, such as SEM, and item response modeling, could better estimate the relationships between latent constructs such as emotional labor types, transformational leadership, and psychological well-being. In contrast to OLS regression, partial least squares SEM is preferable for accommodating measurement errors and allows the simultaneous modeling of various endogenous constructs. Future research should also explore the association between firefighters’ emotional labor and critical work-related outcomes, including organizational citizenship behavior, performance, burnout, and insomnia. This exploration would deepen our understanding of surface and deep acting within public health management. Another important research avenue involves considering the confounding factors that might directly affect psychological well-being or interact with emotional labor, such as psychological capital, organizational climate, psychological empowerment, and person–organization fit. These factors are crucial for fully interpreting the findings of our study. Finally, our study’s external validity is constrained, as all participants were firefighters from South Korea. To broaden these findings, future research should encompass diverse industries such as hospitality, tourism, airlines, and police work.

## Figures and Tables

**Table 1:** Descriptive statistics.

Variable	Mean	S.D.	Min.	Max.
Psychological well-being	3.228	0.414	1.833	4
Surface acting	2.462	0.791	1	5
Deep acting	2.834	0.827	1	5
Transformational leadership	3.620	0.700	1	5
Organizational fairness	3.521	0.783	1	5
Organizational support	3.672	0.769	1	5
Gender (Female = 1)	0.138	0.345	0	1
Age	2.341	0.967	1	4
Job grade	2.635	1.395	1	7
Education	2.319	0.828	1	4
Tenure	1.591	0.800	1	4
Marital status (Married = 1)	0.619	0.486	0	1

Note. N = 1453; S.D. = standard deviation.

**Table 2:** Results of confirmatory factor analyses.

Model	$\chi^{2}$	d.f.	RMSEA	CFI	TLI	SRMR
Six-factor model	2991.82 ***	390	0.06	0.92	0.91	0.07
Five-factor model (SA and DA combined)	5847.78 ***	395	0.09	0.82	0.81	0.09
Four-factor model (SA, DA, and OF combined)	8124.26 ***	399	0.11	0.75	0.73	0.12
Three-factor model (SA, DA, OF, and OS combined)	12192.31 ***	402	0.14	0.62	0.59	0.14
Two-factor model (SA, DA, OF, OS, and TFL combined)	16822.26 ***	433	0.16	0.49	0.48	0.12
One-factor model	19039.94 ***	434	0.17	0.42	0.37	0.13

Note. *** *p* < 0.001; d.f. = degree of freedom; SA = surface acting; DA = deep acting; OF = organizational fairness; OS = organizational support; TFL = transformational leadership.

**Table 3:** Correlation coefficients, Cronbach’s α, and AVE.

	(1)	(2)	(3)	(4)	(5)	(6)	(7)	(8)	(9)	(10)	(11)	(12)
(1)	1.00											
(2)	−0.50 ***	1.00										
(3)	−0.11 ***	0.17 ***	1.00									
(4)	0.40 ***	−0.32 ***	0.02	1.00								
(5)	0.34 ***	−0.31 ***	0.06 *	0.48 ***	1.00							
(6)	0.43 ***	−0.31 ***	0.04 ^†^	0.55 ***	0.50 ***	1.00						
(7)	−0.09 ***	0.06 *	−0.09 ***	−0.07 **	−0.03	−0.11	1.00					
(8)	−0.01	−0.13 ***	0.14 ***	0.05 *	0.11 ***	0.12 ***	−0.15 ***	1.00				
(9)	0.01	−0.12 ***	0.14 ***	0.08 **	0.12 ***	0.13 ***	−0.11 ***	0.81 ***	1.00			
(10)	−0.03	0.04	−0.01	−0.06 *	−0.04	−0.08 ***	0.13 ***	−0.05 *	−0.08 **	1.00		
(11)	0.02	−0.13 ***	0.18 ***	0.12 ***	0.14 ***	0.15 **	−0.12 ***	0.81 ***	0.84 ***	−0.13 ***	1.00	
(12)	0.05	−0.09 ***	0.07 **	0.00	0.06*	0.06 ***	−0.06 *	0.60 ***	0.63 ***	0.00	0.50 ***	1.00
Cronbach’s alpha	0.82	0.86	0.90	0.93	0.96	0.93 *	N.A.	N.A.	N.A.	N.A.	N.A.	N.A.
AVE	0.61	0.67	0.76	0.72	0.86	0.77	N.A.	N.A.	N.A.	N.A.	N.A.	N.A.

Note. ^†^ *p* < 0.1; * *p* < 0.05; ** *p* < 0.01; *** *p* < 0.001; (1) = psychological well-being; (2) = surface acting; (3) = deep acting; (4) = transformational leadership; (5) = organizational fairness; (6) = organizational support; (7) = gender; (8) = age; (9) = job grade; (10) = education; (11) = tenure; (12) = marital status; N.A. = not applicable.

**Table 4:** OLS regression results for the hypothesized relationships.

	Model 1	Model 2	Model 3
	β	β	β
	(S.E.)	(S.E.)	(S.E.)
Gender (Female = 1)	−0.060 *	−0.059 *	−0.060 *
	(0.027)	(0.024)	(0.024)
Age	–0.037 ^†^	−0.050 **	−0.048 **
	(0.019)	(0.048)	(0.017)
Job grade	−0.013	−0.015	−0.015
	(0.015)	(0.014)	(0.013)
Education	0.000	0.003	0.003
	(0.012)	(0.011)	(0.011)
Tenure	−0.010	0.002	0.001
	(0.025)	(0.024)	(0.024)
Marital status (Married = 1)	0.100 ***	0.087 ***	0.087 ***
	(0.026)	(0.023)	(0.023)
Organizational fairness	0.064 ***	0.034 **	0.039 **
	(0.015)	(0.014)	(0.014)
Organizational support	0.139 ***	0.112 ***	0.114 ***
	(0.016)	(0.015)	(0.015)
Transformational leadership	0.125 ***	0.088 ***	−0.059
	(0.017)	(0.016)	(0.047)
Surface acting		−0.193 ***	−0.314 ***
		(0.013)	(0.055)
Deep acting		−0.024 *	−0.115 *
		(0.011)	(0.056)
Surface acting × Transformational leadership			0.033 *
			(0.014)
Deep acting × Transformational leadership			0.023 ^†^
			(0.014)
Constant	2.126 ***	3.024 ***	3.554 ***
	(0.068)	(0.085)	(0.184)
R-squared	0.246	0.370	0.375

Note. ^†^ *p* < 0.1; * *p* < 0.05; ** *p* < 0.01; *** *p* < 0.001; S.E. = robust standard error; N = 1453.

## Data Availability

Data will be made available on request.

## References

[1] Ryu H.-Y., Hyun D.-S., Jeung D.-Y., Kim C.-S., Chang S.-J. (2020). Organizational Climate Effects on the Relationship between Emotional Labor and Turnover Intention in Korean Firefighters. Saf. Health Work..

[2] Back C.-Y., Hyun D.-S., Chang S.-J., Jeung D.-Y. (2023). Trauma Exposure and Suicidal Ideation among Korean Male Firefighters: Examining the Moderating Roles of Organizational Climate. Saf. Health Work..

[3] Serrano-Ibáñez E.R., Corrás T., Del Prado M., Diz J., Varela C. (2023). Psychological Variables Associated With Post-Traumatic Stress Disorder in Firefighters: A Systematic Review. Trauma Violence Abus..

[4] Jeung D.-Y., Chang S.-J. (2021). Moderating Effects of Organizational Climate on the Relationship between Emotional Labor and Burnout among Korean Firefighters. Int. J. Environ. Res. Public Health.

[5] Payne N., Kinman G. (2019). Job Demands, Resources and Work-Related Well-Being in UK Firefighters. Occup. Med..

[6] Pugh S.D., Groth M., Hennig-Thurau T. (2011). Willing and Able to Fake Emotions: A Closer Examination of the Link between Emotional Dissonance and Employee Well-Being. J. Appl. Psychol..

[7] Rogers M.E., Creed P.A., Searle J. (2014). Emotional Labour, Training Stress, Burnout, and Depressive Symptoms in Junior Doctors. J. Vocat. Educ. Train..

[8] Zapf D. (2002). Emotion Work and Psychological Well-Being: A Review of the Literature and Some Conceptual Considerations. Hum. Resour. Manag. Rev..

[9] Sciotto G., Pace F. (2022). The Role of Surface Acting in the Relationship between Job Stressors, General Health and Need for Recovery Based on the Frequency of Interactions at Work. Int. J. Environ. Res. Public Health.

[10] Grandey A.A. (2000). Emotional Regulation in the Workplace: A New Way to Conceptualize Emotional Labor. J. Occup. Health Psychol..

[11] Hobfoll S.E. (1989). Conservation of Resources: A New Attempt at Conceptualizing Stress. Am. Psychol..

[12] Du Y., Wang Z. (2021). How Does Emotional Labor Influence Voice Behavior? The Roles of Work Engagement and Perceived Organizational Support. Sustainability.

[13] Hur W.-M., Han S.-J., Yoo J.-J., Moon T.W. (2015). The Moderating Role of Perceived Organizational Support on the Relationship between Emotional Labor and Job-Related Outcomes. Manag. Decis..

[14] Chi N.-W., Wang I.-A. (2018). The Relationship between Newcomers’ Emotional Labor and Service Performance: The Moderating Roles of Service Training and Mentoring Functions. Int. J. Hum. Resour. Manag..

[15] Aziz S., Widis A., Wuensch K. (2018). The Association between Emotional Labor and Burnout: The Moderating Role of Psychological Capital. Occup. Health Sci..

[16] Katou A.A., Koupkas M., Triantafillidou E. (2022). Job Demands-Resources Model, Transformational Leadership and Organizational Performance: A Multilevel Study. Int. J. Product. Perform. Manag..

[17] Hülsheger U.R., Schewe A.F. (2011). On the Costs and Benefits of Emotional Labor: A Meta-Analysis of Three Decades of Research. J. Occup. Health Psychol..

[18] Choi M.Y. (2021). Mental and Physical Factors Influencing Wellbeing among South Korean Emergency Workers. Int. J. Environ. Res. Public Health.

[19] Le H., Gopalan N., Lee J., Kirige I., Haque A., Yadav V., Lambropoulos V. (2023). Impact of Work and Non-Work Support on Employee Well-Being: The Moderating Role of Perceived Organizational Support. Sustainability.

[20] Hochschild A.R. (1979). Emotion Work, Feeling Rules, and Social Structure. Am. J. Sociol..

[21] Arvey R.W., Renz G.L., Watson T.W. (1998). Emotionality and Job Performance: Implications for Personnel Selection. Research in Personnel and Human Resources Management.

[22] Yin H., Wang W., Huang S., Li H. (2018). Psychological Capital, Emotional Labor and Exhaustion: Examining Mediating and Moderating Models. Curr. Psychol..

[23] Kammeyer-Mueller J.D., Rubenstein A.L., Long D.M., Odio M.A., Buckman B.R., Zhang Y., Halvorsen-Ganepola M.D.K. (2013). A Meta-Analytic Structural Model of Dispositonal Affectivity and Emotional Labor. Pers. Psychol..

[24] Park H., Kim J.I., Min B., Oh S., Kim J.-H. (2019). Prevalence and Correlates of Suicidal Ideation in Korean Firefighters: A Nationwide Study. BMC Psychiatry.

[25] Imose R.A., Finkelstein L.M. (2018). A Multilevel Theoretical Framework Integrating Diversity and Emotional Labor. Group Organ. Manag..

[26] Van Gelderen B.R., Konijn E.A., Bakker A.B. (2017). Emotional Labor among Police Officers: A Diary Study Relating Strain, Emotional Labor, and Service Performance. Int. J. Hum. Resour. Manag..

[27] Rubaca U., Majid Khan M. (2021). The Impact of Perceived Organizational Support and Job Resourcefulness on Supervisor-Rated Contextual Performance of Firefighters: Mediating Role of Job Satisfaction. J. Contingencies Crisis Manag..

[28] Lee J.J., Ok C.M. (2014). Understanding Hotel Employees’ Service Sabotage: Emotional Labor Perspective Based on Conservation of Resources Theory. Int. J. Hosp. Manag..

[29] Brotheridge C.M., Grandey A.A. (2002). Emotional Labor and Burnout: Comparing Two Perspectives of “People Work”. J. Vocat. Behav..

[30] Lee G. (2015). Korean Emotional Laborers’ Job Stressors and Relievers: Focus on Work Conditions and Emotional Labor Properties. Saf. Health Work..

[31] Ito J.K., Brotheridge C.M. (2003). Resources, Coping Strategies, and Emotional Exhaustion: A Conservation of Resources Perspective. J. Vocat. Behav..

[32] Lee Y.H. (2019). Emotional Labor, Teacher Burnout, and Turnover Intention in High-School Physical Education Teaching. Eur. Phys. Educ. Rev..

[33] Hill N.S., Zhang H., Zhang X., Ziwei Y. (2020). The impact of surface and deep acting on employee creativity. Creat. Res. J..

[34] Gross J.J. (2002). Emotion Regulation: Affective, Cognitive, and Social Consequences. Psychophysiology.

[35] Hur W.-M., Rhee S.-Y., Ahn K.-H. (2016). Positive Psychological Capital and Emotional Labor in Korea: The Job Demands-Resources Approach. Int. J. Hum. Resour. Manag..

[36] Nguyen H., Groth M., Johnson A. (2016). When the Going Gets Tough, the Tough Keep Working: Impact of Emotional Labor on Absenteeism. J. Manag..

[37] Correia Leal C., Ferreira A.I., Carvalho H. (2023). “Hide Your Sickness and Put on a Happy Face”: The Effects of Supervision Distrust, Surface Acting, and Sickness Surface Acting on Hotel Employees’ Emotional Exhaustion. J. Organ Behav..

[38] Brotheridge C.M., Lee R.T. (2002). Testing a Conservation of Resources Model of the Dynamics of Emotional Labor. J. Occup. Health Psychol..

[39] Goldberg L.S., Grandey A.A. (2007). Display Rules versus Display Autonomy: Emotion Regulation, Emotional Exhaustion, and Task Performance in a Call Center Simulation. J. Occup. Health Psychol..

[40] Xu S., Martinez L.R., Lv Q. (2017). Full Article: Explaining the Link Between Emotional Labor and Turnover Intentions: The Role of In-Depth Communication. Int. J. Hosp. Tour. Adm..

[41] Wang I.-A., Lin S.-Y., Chen Y.-S., Wu S.-T. (2022). The Influences of Abusive Supervision on Job Satisfaction and Mental Health: The Path through Emotional Labor. Pers. Rev..

[42] Bass B.M. (1985). Leadership and Performance beyond Expectations.

[43] Wang G., Oh I.-S., Courtright S.H., Colbert A.E. (2011). Transformational Leadership and Performance Across Criteria and Levels: A Meta-Analytic Review of 25 Years of Research. Group Organ. Manag..

[44] Judge T.A., Piccolo R.F. (2004). Transformational and Transactional Leadership: A Meta-Analytic Test of Their Relative Validity. J. Appl. Psychol..

[45] Deng C., Gulseren D., Isola C., Grocutt K., Turner N. (2023). Transformational Leadership Effectiveness: An Evidence-Based Primer. Hum. Resour. Dev. Int..

[46] Hentrich S., Zimber A., Garbade S.F., Gregersen S., Nienhaus A., Petermann F. (2017). Relationships between Transformational Leadership and Health: The Mediating Role of Perceived Job Demands and Occupational Self-Efficacy. Int. J. Stress Manag..

[47] Demerouti E., Bakker A.B., Nachreiner F., Schaufeli W.B. (2001). The Job Demands-Resources Model of Burnout. J. Appl. Psychol..

[48] Bakker A.B., Demerouti E., Sanz-Vergel A.I. (2014). Burnout and Work Engagement: The JD–R Approach. Annu. Rev. Organ. Psychol. Organ. Behav..

[49] Alarcon G.M. (2011). A Meta-Analysis of Burnout with Job Demands, Resources, and Attitudes. J. Vocat. Behav..

[50] Xanthopoulou D., Bakker A.B., Dollard M.F., Demerouti E., Schaufeli W.B., Taris T.W., Schreurs P.J. (2007). When Do Job Demands Particularly Predict Burnout? The Moderating Role of Job Resources. J. Manag. Psychol..

[51] Schaufeli W.B. (2015). Engaging Leadership in the Job Demands-Resources Model. Career Dev. Int..

[52] Berger R., Czakert J.P., Leuteritz J.-P., Leiva D. (2019). How and When Do Leaders Influence Employees’ Well-Being? Moderated Mediation Models for Job Demands and Resources. Front. Psychol..

[53] Díaz-Fúnez P.A., Salvador-Ferrer C.M., García-Tortosa N., Mañas-Rodríguez M.A. (2021). Are Job Demands Necessary in the Influence of a Transformational Leader? The Moderating Effect of Role Conflict. Int. J. Environ. Res. Public Health.

[54] Holstad T.J., Korek S., Rigotti T., Mohr G. (2014). The Relation between Transformational Leadership and Follower Emotional Strain: The Moderating Role of Professional Ambition. Leadership.

[55] Inceoglu I., Thomas G., Chu C., Plans D., Gerbasi A. (2018). Leadership Behavior and Employee Well-Being: An Integrated Review and a Future Research Agenda. Leadersh. Q..

[56] Cheung F., Tang C.S., Tang S. (2011). Psychological Capital as a Moderator between Emotional Labor, Burnout, and Job Satisfaction among School Teachers in China. Int. J. Stress Manag..

[57] Wu T., Hu C. (2013). Abusive Supervision and Subordinate Emotional Labor: The Moderating Role of Openness Personality. J. Appl. Soc. Pyschol..

[58] Nixon A.E., Yang L., Spector P.E., Zhang X. (2011). Emotional Labor in China: Do Perceived Organizational Support and Gender Moderate the Process?. Stress Health.

[59] Nilsson K.W., Leppert J., Simonsson B., Starrin B. (2009). Sense of Coherence (SOC) and Psychological Well-Being (GHQ): Improvement with Age. J. Epidemiol. Community Health.

[60] Archangelidi O., Mentzakis E. (2018). Body-Weight and Psychological Well-Being in the UK General Population. J. Public Health.

[61] Goldberg D.P., Williams P. (1988). A User’s Guide to the General Health Questionnaire.

[62] House R.J., Dansereau F., Yammarino F.J. (1998). Appendix: Measures and Assessments for the Charismatic Leadership Approach: Scales, Latent Constructs, Loadings, Cronbach Alphas, Interclass Correlations. Leadership: The Multiple Level Approaches Contemporary and Alternative.

[63] Folger R.G., Folger R., Cropanzano R. (1998). Organizational Justice and Human Resource Management.

[64] Leventhal G.S., Berkowitz L., Walster W. (1976). The Distribution of Rewards and Resources in Groups and Organizations. Advances in Experimental Social Psychology.

[65] Eisenberger R., Huntington R., Hutchison S., Sowa D. (1986). Perceived Organizational Support. J. Appl. Psychol..

